# In Memoriam: Em. Professor Dr. Anders Lunderquist (1925–2023)

**DOI:** 10.5334/jbsr.3631

**Published:** 2024-05-10

**Authors:** Robert Ferdinand Dondelinger

**Affiliations:** 1 University of Liège, Liège, Belgium

On December 12, 2023, a giant of interventional radiology passed away. Anders Lunderquist was born in Lycksele, North of Umea, Sweden, in 1925. He accomplished medical studies at the University of Lund and became involved in the development of the world’s first clinically usable artificial kidney, invented by Professor Nils Alwall in 1946. However, Anders did not pursue a career in nephrology. He turned to radiology, starting residency training at the county hospital in Kalmar, a small city facing the island of Öland. Anders, who grew up in a forest environment, became attached to the untamed insular beauty, and he enjoyed spending holidays with his wife in their summerhouse on Öland.

Under the guidance of Professor Olle Olson, who shaped the new department of radiology at the University Hospital of Lund and fostered radiological research, Anders committed to diagnostic angiography, still in its adolescence in those days. In 1965, he presented a PhD dissertation on ‘*Angiography in carcinoma of the pancreas.’* Following the post-World War I avant-garde work of the Portuguese school of angiography headed by Antonio Egas Moniz, Lund evolved as a center of excellence in clinical angiography. Erik Boijsen, 3 years elder than Anders, also pioneered selective visceral catheter angiography at the same department. For many years, angiographists flocked to Lund from all over the world to get exposure to angiographic techniques under the teaching of Erik and Anders. Both radiologists were among the driving forces of the European College of Angiography. This society counted 75 members, mainly originating from Northern Europe, and merged with the European Society of Cardiovascular Radiology to form a new society called the ‘Cardiovascular and Interventional Radiological Society of Europe,’ in Vienna in 1985. The radiology department of Lund University produced eminent academic Swedish angiographists, including Leif Ekelund, Ulf Tylen, and Jan Göthlin. Many angiographists from Europe and overseas were trained in Lund. The clinical and surgical activities were a stimulating environment for angiographists, largely supported by the research conducted by the visceral surgeons Philip Sandblom, Stig Bengmark, and Ingemar Ihse, promoting hepatobiliary and pancreatic surgery. Prime-quality diagnostic angiography was crucial for creative visceral surgery in the era preceding cross-sectional imaging.

Anders was one of the most skilled angiographists in his era. He adopted a pragmatic approach to radiological–clinical problems and practiced patient-oriented radiology putting the patient always first. Anders handed his business card to the patients after the completion of the examination, encouraging personal contact. After the daily routine, Anders prepared the radiographic films for the regular multidisciplinary case discussion of early next day. In 1974, Lunderquist and Vang published the results of percutaneous transhepatic embolization of esophageal varices fed by the left gastric vein. This innovational clinical research paved the way for further interventions in the portal vein system and can be considered as the early stage of radiological intrahepatic porto-systemic shunts. Anders’ work was also innovative in nonvascular intervention. Wiechel, a surgeon at Karolinska Hospital in Stockholm, had accumulated a large experience in percutaneous needle opacification of the intrahepatic bile ducts, which led to Sven Ivar Seldinger’s PhD thesis on ‘*Percutaneous cholangiography’* at Karolinska Institute in 1966. Anders further refined Wiechel’s percutaneous transhepatic bile duct cannulation and, consequently, biliary drainage techniques. The concept of the *stiff Lunderquist guide wire* remains a milestone in the armamentarium of transhepatic biliary drainage. The device is applied in other interventional procedures as well, especially whenever strong pushing forces must be transmitted over this. Similarly, the design of the *Ring-Lunderquist* biliary drainage catheter is still popular. After retirement, Anders continued research activities, ultimately conceiving an experimental technique of retrograde rapid cooling of the brain without affecting the body temperature in 2006.

**Figure:**
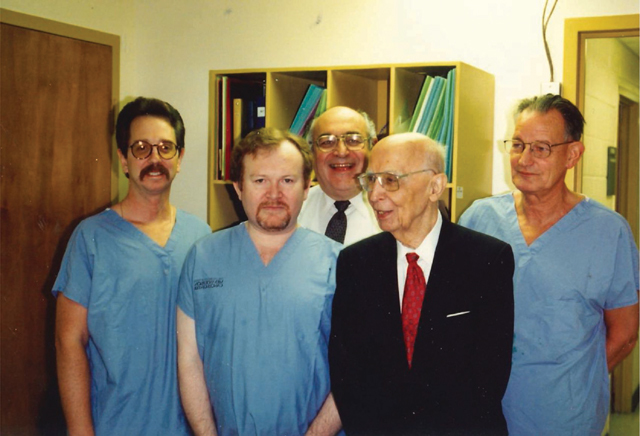
Instructors of a ‘Lunderquist’ Hands-on Course in Interventional Radiology at MD Anderson Cancer Center, Houston, Texas, in 1997. From left to right : Kenneth Wright, Robert F. Dondelinger, Sidney Wallace, Cesare Gianturco, and Anders Lunderquist.

Anders was an exquisitely talented teacher. He tailored 1-week hands-on courses in interventional radiology based on animal models. Vascular, biliary, and ureteral strictures were operatively created in pigs to produce pathological obstruction for treatment purposes. These courses run for many years in the same format in Lund-Malmö, at the MD Anderson Cancer Center, Houston, Texas, supervised by Sidney Wallace, and at the University of Liège, Belgium, always with the same team of instructors. This practical teaching, which was innovative for its time, attracted hundreds of students from many countries.

Anders Lunderquist authored about 200 scientific papers. He earned honoris causa doctor titles from several universities and was a visiting professor in many countries, traveling most often with his wife, Rut, who was a nurse at the University Hospital of Lund. He was awarded with the gold medal of the American Society of Interventional Radiology in 1998 and the gold medal of the Cardiovascular and Interventional Radiological Society of Europe the following year. Anders was an honorary member of the Belgian Society of Radiology.

Anders is survived by his daughter Marianne and his grandson Anders.

